# Whole body bone scintigraphy in tenofovir-related osteomalacia: a case report

**DOI:** 10.4076/1752-1947-3-8136

**Published:** 2009-07-22

**Authors:** Antonio Di Biagio, Raffaella Rosso, Patrizia Monteforte, Rodolfo Russo, Guido Rovetta, Claudio Viscoli

**Affiliations:** 1 Department of Infectious Diseases, San Martino Hospital, University of Genoa, Genoa 16132, Italy; 2 Department of Endocrinology and Metabolism Sciences, University of Genoa, Genoa 16132, Italy; 3 Nephrology Division, Department of Internal Medicine, San Martino Hospital, University of Genoa, Genoa 16132, Italy

## Abstract

**Introduction:**

Tenofovir disoproxil fumarate (Viread^®^) is the only nucleotide reverse transcriptase inhibitor currently approved for the treatment of HIV. It is frequently prescribed not only for its efficacy but also for its decreased side effect profile compared with other nucleotide analogs. In addition, it is now increasingly recognized as a cause of acquired Fanconi's syndrome in individuals with HIV.

**Case presentation:**

We describe a 48-year-old woman infected with HIV, with chronic renal insufficiency, who developed Fanconi's syndrome after inclusion of tenofovir disoproxil fumarate in her antiretroviral therapy. A whole body bone scintigraphy was performed, revealing an abnormal distribution of radiotracer uptake, with characteristic changes compatible with osteomalacia. All symptoms disappeared after tenofovir discontinuation and mineral supplementation. No other explanation for the sudden and complete resolution of the bone disease was found.

**Conclusion:**

The case highlights the role of whole body bone scintigraphy in the diagnosis of tenofovir-related osteomalacia.

## Introduction

Tenofovir disoproxil fumarate (TDF) is an oral prodrug of tenofovir, a nucleotide reverse transcriptase inhibitor (NRTI). Because of its favorable resistance profile and its activity against HIV-1 strains, TDF is widely used as part of highly active antiretroviral therapy (HAART). TDF is rapidly hydrolyzed and is mainly eliminated unchanged by the kidney, by a combination of glomerular filtration and active tubular secretion [1]. Nephrotoxicities owing to TDF have been reported over the past few years. In particular, TDF is capable of causing a Fanconi-like syndrome with renal phosphate wasting and concomitant osteomalacia [2]-[5].

Since the advent of HAART, multiple epidemiologic studies have shown that osteopenia and osteoporosis are common among patients with HIV infection [6]. Osteoporosis is well-documented by means of bone densitometry (DEXA), while bone scintigraphy is useful for identifying fractures. In patients with HIV, osteomalacia has been documented in only a few cases by DEXA, it appears as bone demineralization and its reversibility has been described [7,8].

A whole body bone scintigraphy, in order to evaluate patients who are HIV-negative with bone disorders, is well-defined [9]. We report a severe case of osteomalacia in a woman infected with HIV, diagnosed by bone scan and resolved after TDF discontinuation.

## Case presentation

A 48-year-old Caucasian woman was diagnosed with HIV-1 infection in 1992. Past medical history included primary amenorrhea (1970), psoriasis (1974), detection of anti-hepatitis C virus (HCV) antibodies and psoriasis-related mild chronic renal insufficiency (1995). In May 2002, she was diagnosed with osteoporosis, based on DEXA, and started therapy with bisphosphonates (disodium clodronate 100 mg intramuscular/die) associated with inhibitor COX-2 (celecoxib 200 mg/die). At the same time, renal ecotomography showed reduced renal size (right kidney 89 × 40 × 43 mm; left kidney 86 × 37 × 49 mm) and parenchymal thickness, but regular morphology. In October 2002, she switched from disodium clodronate to raloxifene (60 mg/die). She was on HAART since 1997: zidovudine 300 mg twice a day (bid) plus lamivudine 150 mg bid plus indinavir 800 mg three times a day from July 1997 to July 2001, changed for simplification; zidovudine 300 mg bid plus lamivudine 150 mg bid plus efavirenz 600 mg once daily (qd) from July 2001 to December 2001, stopped for zidovudine-related anemia, stavudine 30 mg bid plus lamivudine 150 mg bid plus efavirenz 600 mg qd from December 2001 to May 2002, stopped for paresthesia; finally, because of good immunological restoration, she had a lymphocyte T CD4+-guided structured antiretroviral treatment interruption from May 2002 to February 2004.

During the off-therapy period, her serum creatinine level had a peak of 1.8 mg/dl (normal range: 0.5-1.3), and it was 1.7 mg/dl (estimated clearance creatinine 59.4 ml/min) just before starting the new tenofovir-containing regimen. On November 2002, her HCV-RNA concentration was high (3.05 × 10^5^ copies/ml, Amplicore HCV monitor assays: Roche Diagnostic System, USA), while serum transaminase levels were stable (AST 57 U/l; ALT 70 U/l) and within twice the normal range (0-40 UI/l). The patient refused to undergo a liver biopsy.

On February 2004, a new qd regimen including tenofovir 300 mg qd, plus lamivudine 300 mg qd, plus efavirenz 600 mg qd was started; her CD4+ cell count and viral load were 267 cells/mL (21%) and 30000 copies/mL, respectively.

Baseline renal function was mildly impaired: serum alkaline phosphate level was 378 U/l (normal range: 98-280) and urinalysis showed urine protein 1 g/dl, urine glucose 5 g/dl, urine pH6 and traces of hemoglobin.

After four weeks, the patient was admitted to our out-patient clinic for her follow-up visit, and she referred mild bone pain in the proximal area of her tibias and in her ankles; the serum creatinine level at this time was 1.7 mg/dl.

Two weeks later, the patient's bone pain had worsened substantially, especially in her lower-limbs and chest, with myalgia and difficulties in staying upright. She also complained of increasing fatigue and polyuria.

Her laboratory test results revealed serum creatinine level 1.8 mg/dl, alkaline phosphatase 1247 U/I, phosphate 1.6 mg/dl (normal values: 2.5-4.5), potassium 3.1 mEq/litre (normal value: 3.5-5), bicarbonate 11.30 mmol/litre (normal values 22.00-26.00). Urinalysis demonstrated urine glucose >10.0 g/litre, urine pH 6, protein 1.0 g/litre, and traces of ketones. The 24-hour urine collection showed a level of urinary potassium of 1.20 mEq/litre.

**Figure 1:**
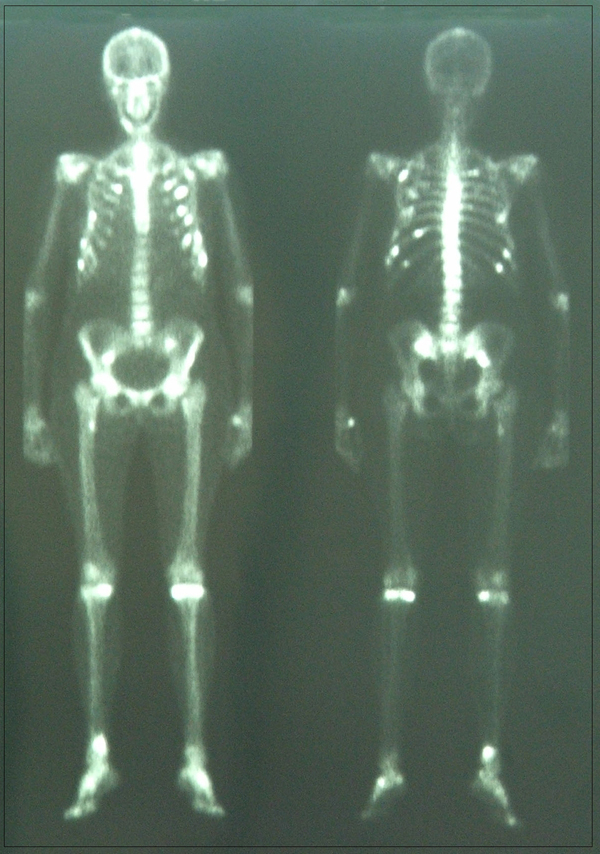
**Whole body bone scintigraphy shows multiple foci of increased radiotracer uptake in the rib cage, the lumbar spine, the sacroiliac region, the bilateral knee and the right tibiotarsus**.

Fanconi's syndrome was suspected in this patient given the presence of hypokalemia, hypophoshatemia, glucosuria and proteinuria. Despite a very good response to HAART (CD4+ cells count 373 cell/mm^3^ (32%) and viral load <50 copies/mL), tenofovir was switched to abacavir 300 mg bid. At that time, radiological evaluation included a skeletal bone scan: it showed costal and vertebral multiple hypercaptations, as well as pelvic hypercaptations of bone tracer, indicating areas of increased bone turnover. The pelvis bone activity was present in locations typical for osteomalacia (Figure 1). Calcitriol regimen, at the recommended doses (50 mcg/die), was started and continued for a year.

**Figure 2:**
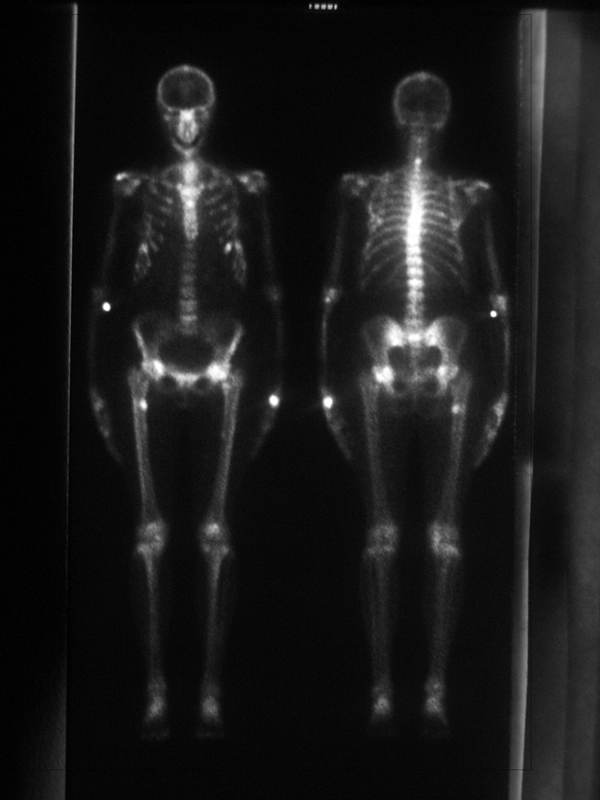
**Repeat scan one year after tenofovir sparing-regimen shows largely disappeared focal lesions**.

Gradually, bone pains and arthralgia improved, and serum alkaline phosphatase normalized. Serum creatinine, urine protein and urine glucose diminished to 1.7 mg/dl, 1.0 g/litre and 5.0 g/litre, respectively; while serum phosphate and potassium increased to 2.4 mg/dL and 3.3 mEq/litre, respectively. After one year, the patient again underwent a bone scintigraphy, which showed the complete absence of hypercaptation (Figure 2).

## Discussion

HAART can readily achieve long-term remission of HIV disease, but it can also have both short- and long-term adverse events [10]. The patient described developed acute renal failure, during TDF-based therapy. However, renal involvement could be explained by HCV infection alone, although we cannot rule out tenofovir as the primary cause. Indeed, the development of nephrotoxicity has a temporal relationship with TDF administration. Moreover, disability was progressively reversed by drug withdrawal and pharmacological interventions. In the differential diagnosis of Fanconi's syndrome, we have also considered other pathologies, such as myeloma, amyloidosis, Sjögren's syndrome, vitamin D deficiency and antibiotic use, but its appearance seemed more related to the combination of chronic renal disease, renal tubular acidosis and nephrotoxicity, possibly secondary to TDF medications.

Osteomalacia is a part of Fanconi's syndrome, and we made a diagnosis of hypophosphataemic osteomalacia because of the patient's lowered serum phosphate level and a rising alkaline phosphatase level, associated with bone pain and typical areas of scintigraphic pelvic activity.

Fanconi's syndrome occurs as a loss of proximal tubular function resulting in its failure to reabsorb various substances, among which glucose, bicarbonate, phosphates, uric acid, potassium, sodium and amino acids, with subsequent loss of these in the urine. The syndrome is defined by a hypokalemic, metabolic acidosis with hypophosphatemia and glucosuria; however, the presence of any combination of these features can occur when the proximal tubule is affected.

Unfortunately, we did not evaluate vitamin D levels, which would have been useful to confirm the diagnosis; in addition, the patient refused a bone biopsy.

Skeletal scintigraphy can be of great value in the diagnosis and evaluation of therapy of many benign bone disorders. In our patient we used this technique in order to diagnose and monitor her severe osteomalacia. However, skeletal scintigraphy has a number of applications, and many metabolic bone disorders characterized by altered blood flow or osteoblastic reaction may be readily identified by the procedure. In contrast to metastatic disease where focal abnormalities are characteristically seen, the bone scan diagnosis of metabolic bone disease generally depends upon recognition of a generalized increase. This bone scan appearance in osteomalacia strongly suggests the presence of a metabolic bone disorder because of a generalized increase in the tracer uptake in the skeleton producing "super scan" with nonvisualisation of kidneys. Since in severe osteomalacia, pseudofractures are common, increased focal bone uptake of radiopharmaceutical is also found [9].

## Conclusions

We have described a patient with HIV/HCV-infection and psoriasis-related chronic renal failure. Bone scintigraphy has proven to be useful for the diagnosis of osteomalacia, a possible consequence of Fanconi's syndrome that can occur with TDF therapy.

It is acknowledged that the subject reported in this case report was antiretroviral treatment-experienced and consequently received TDF as part of a salvage regimen; these type of patients, often also HCV co-infected, may have been subjected to different antiretrovirals prescribing patterns which may add to the clinical presentation described.

A whole body bone scintigraphy can be indicated in patients on TDF with bone and joint pain. The bone scan pattern in typical osteomalacia can be focal, similar to osseous metastases, or diffuse, as in our patient.

Mineral supplementation and cessation of TDF appear to be the treatment of choice in patients who show features of the Fanconi's syndrome. Patients with underlying renal abnormality, or who show features of the Fanconi's syndrome during follow-up, should discontinue TDF; equally in patients who develop bone pain or myopathy on TDF treatment, hypophosphatemia should be looked for and treated and the drug stopped.

Adverse effects have been reported with virtually all antiretroviral drugs and are the most common reasons for switching or discontinuation of therapy and for medication nonadherence: a better understanding is of interest not only for HIV specialists as they try to optimize therapy, but also for other physicians who provide care for patients infected with HIV.

## Abbreviations

COX: cyclooxygenase; HAART: Highly Active Anti-Retroviral Therapy; HIV: Human Immunodeficiency Virus.

## Consent

Written informed consent was obtained from the patient for publication of this case report and any accompanying images. A copy of the written consent is available for review by the Editor-in-Chief of this journal.

## Competing interests

The authors declare that they have no competing interests.

## Authors' contributions

AD, RaR and CV revised the article for intellectual content and helped to draft the manuscript. RoR interpreted the renal results. PM and GD supervised the acquisition process and interpreted the scintigraphic images. All authors read and approved the final manuscript.
